# Preventive Effect of Canstatin against Ventricular Arrhythmia Induced by Ischemia/Reperfusion Injury: A Pilot Study

**DOI:** 10.3390/ijms22031004

**Published:** 2021-01-20

**Authors:** Akira Sugiyama, Yurie Shimizu, Muneyoshi Okada, Kosuke Otani, Hideyuki Yamawaki

**Affiliations:** Laboratory of Veterinary Pharmacology, School of Veterinary Medicine, Kitasato University, Higashi 23 Bancho 35-1, Towada City, Aomori 034-8628, Japan; dv17003@st.kitasato-u.ac.jp (A.S.); vm14065f@st.kitasato-u.ac.jp (Y.S.); otani@vmas.kitasato-u.ac.jp (K.O.); yamawaki@vmas.kitasato-u.ac.jp (H.Y.)

**Keywords:** arrhythmia, calcium overload, canstatin, ischemia/reperfusion, reactive oxygen species

## Abstract

Ventricular arrhythmia induced by ischemia/reperfusion (I/R) injury is a clinical problem in reperfusion therapies for acute myocardial infarction. Ca^2+^ overload through reactive oxygen species (ROS) production is a major cause for I/R-induced arrhythmia. We previously demonstrated that canstatin, a C-terminal fragment of type IV collagen α2 chain, regulated Ca^2+^ handling in rat heart. In this study, we aimed to clarify the effects of canstatin on I/R-induced ventricular arrhythmia in rats. Male Wistar rats were subjected to I/R injury by ligating the left anterior descending artery followed by reperfusion. Ventricular arrhythmia (ventricular tachycardia and ventricular fibrillation) was recorded by electrocardiogram. Nicotinamide adenine dinucleotide phosphate oxidase (NOX) activity and ROS production in neonatal rat cardiomyocytes (NRCMs) stimulated with oxygen glucose deprivation/reperfusion (OGD/R) were measured by lucigenin assay and 2′,7′-dichlorodihydrofluorescein diacetate staining, respectively. The H_2_O_2_-induced intracellular Ca^2+^ ([Ca^2+^]_i_) rise in NRCMs was measured by a fluorescent Ca^2+^ indicator. Canstatin (20 µg/kg) inhibited I/R-induced ventricular arrhythmia in rats. Canstatin (250 ng/mL) inhibited OGD/R-induced NOX activation and ROS production and suppressed the H_2_O_2_-induced [Ca^2+^]_i_ rise in NRCMs. We for the first time demonstrated that canstatin exerts a preventive effect against I/R-induced ventricular arrhythmia, perhaps in part through the suppression of ROS production and the subsequent [Ca^2+^]_i_ rise.

## 1. Introduction

Myocardial infarction (MI) is caused by a coronary thrombosis mainly due to the disruption of atherosclerotic plaque [1]. Reperfusion therapies, such as percutaneous coronary intervention and thrombolytic therapy, are established as the most effective therapies for acute MI (AMI) [2]. However, ischemia/reperfusion (I/R) injury, a critical complication of the therapies, is a serious clinical problem associated with a high mortality [3,4]. More than 80% of AMI patients develop arrhythmia, including lethal ventricular tachycardia (VT) and ventricular fibrillation (VF), during the first 48 h after reperfusion [4]. However, there is no effective strategy for preventing I/R injury and subsequent arrhythmia [2].

I/R injury induces reactive oxygen species (ROS) production, which causes Ca^2+^ overload via increasing intracellular Ca^2+^ ([Ca^2+^]_i_) through the regulation of Ca^2+^ handling proteins, such as L-type Ca^2+^ channel (LTCC), Na^+^-Ca^2+^ exchanger (NCX), ryanodine receptor (RyR) and sarcoplasmic reticulum Ca^2+^-ATPase (SERCA), in cardiomyocytes [5,6]. The Ca^2+^ overload leads to early afterdepolarizations (EADs) and delayed afterdepolarizations (DADs), which trigger ventricular arrhythmia [6,7,8]. Verapamil, a non-dihydropyridine Ca^2+^ channel blocker, is widely used as a Vaughan Williams class IV anti-arrhythmic drug [9]. However, the usefulness of the Ca^2+^ channel blockers for the early treatment of AMI is still under discussion, since adverse events have been reported, including the worsening of heart failure [9,10].

Canstatin, a C-terminal fragment of the type IV collagen α2 chain, was originally discovered as an endogenous anti-angiogenic and anti-tumor factor [11]. Canstatin is abundantly expressed in normal rat hearts, the expression of which is decreased in the infarcted area after MI [12,13]. We previously reported that a knockdown of canstatin by injecting a small interference (si)RNA of type IV collagen α2 chain gene (*COL4A2*) in rats induced changes in electrocardiogram (ECG) parameters, such as the shortening of the QT interval and the increasing of the T wave amplitude [14]. Furthermore, in ventricular myocytes from the *COL4A2* siRNA-injected rats, LTCC current was increased, which was reversed by an exogenous canstatin [14]. We also demonstrated that canstatin inhibited the isoproterenol-induced [Ca^2+^]_i_ rise in neonatal rat cardiomyocytes (NRCMs) [15]. Thus, it is suggested that canstatin regulates Ca^2+^ handling in rat heart.

In the present study, we hypothesized that canstatin suppresses ventricular arrhythmia by inhibiting the Ca^2+^ overload induced by I/R injury. To test this hypothesis, we investigated the effects of canstatin on I/R-induced ventricular arrhythmia using an in vivo model of I/R-induced ventricular arrhythmia. We also examined the detailed mechanisms of the effect of canstatin using an in vitro model of I/R injury in NRCMs.

## 2. Results

### 2.1. Canstatin Inhibited I/R-Induced Ventricular Arrhythmia

**Figure 1:**
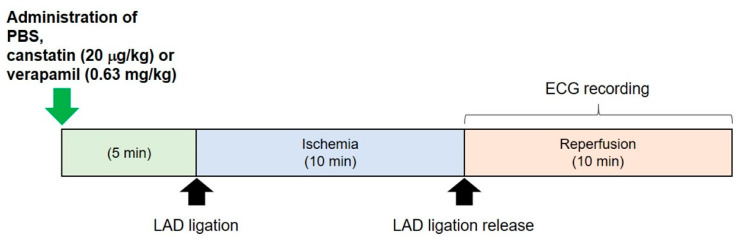
Protocol for the in vivo study of ischemia/reperfusion (I/R)-induced ventricular arrhythmia. Phosphate buffered saline (PBS), canstatin (20 μg/kg) or verapamil (0.63 mg/kg) was intravenously administered to rats. Five minutes after the administration, left anterior descending artery (LAD) ligation was performed (ischemia). Ten minutes after the ligation, it was released (reperfusion). An electrocardiogram (ECG) recording was performed for 10 min after reperfusion.

**Figure 2:**
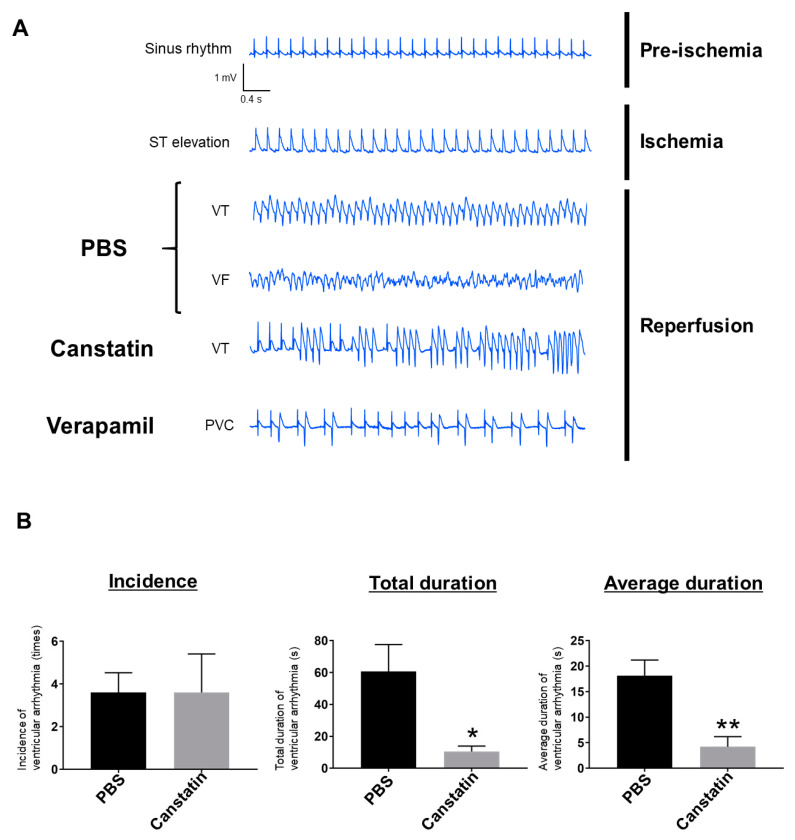
Canstatin inhibited I/R-induced ventricular arrhythmia. PBS, canstatin (20 µg/kg) or verapamil (0.63 mg/kg) was intravenously administered to rats. Five minutes after the administration, I/R injury was induced by ligating LAD for 10 min (ischemia) followed by releasing it (reperfusion). ECG (lead II) was recorded throughout the experiments. (**A**) Representative ECG tracings of sinus rhythm during pre-ischemia, ST elevation during ischemia and arrhythmias in PBS-, canstatin- and verapamil-administered rats during reperfusion were shown. VT: ventricular tachycardia, VF: ventricular fibrillation, PVC: premature ventricular contraction. (**B**) Incidence (left), total duration (middle) and average duration (right) of ventricular arrhythmia (VT and VF) for 10 min after reperfusion were calculated from the ECG tracings and shown as mean ± standard error of the mean (S.E.M.) ((**A**): PBS, Canstatin: *n* = 5, Verapamil: *n* = 3, (**B**): *n* = 5). *, ** *p* < 0.05, 0.01 vs. PBS.

We first examined the effect of canstatin on I/R-induced ventricular arrhythmia in rats (Figure 1 and Figure 2). We confirmed that all rats showed a sinus rhythm during pre-ischemia (Figure 2A) and a significant ST elevation during ischemia (Figure 2A). In all phosphate buffered saline (PBS)-administered rats, VT and VF were observed during reperfusion (Figure 2A). Although VT was observed in 80% (4/5) of canstatin-administered rats, VF was not observed (Figure 2A). In verapamil-administered rats, only premature ventricular contraction was observed (Figure 2A). A quantitative analysis for ventricular arrhythmia (VT and VF) showed that canstatin had no effect on incidence (PBS: 3.6 ± 0.9 times vs. canstatin: 3.6 ± 1.8 ×, *n* = 5), but significantly inhibited the total and average durations of ventricular arrhythmia (total duration—canstatin: 10.5 ± 3.4 s vs. PBS: 60.7 ± 16.9 s, *p* < 0.05; average duration—canstatin: 4.2 ± 2.0 s vs. PBS: 18.2 ± 3.0 s, *p* < 0.01, *n* = 5) (Figure 2B). Verapamil completely suppressed the incidence and duration of ventricular arrhythmia (*n* = 3) (data not shown).

### 2.2. Canstatin Inhibited Oxygen and Glucose Deprivation Followed by Reoxygenation (OGD/R)-Induced Nicotinamide Adenine Dinucleotide Phosphate (NADPH) Oxidase (NOX) Activation in NRCMs

**Figure 3:**
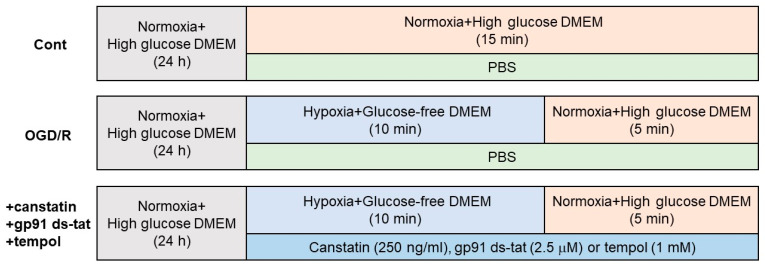
Protocols for oxygen glucose deprivation/reperfusion (OGD/R) stimulation in neonatal rat cardiomyocytes (NRCMs). NRCMs were starved for 24 h in serum-free high-glucose-containing Dulbecco’s modified Eagle’s medium (DMEM) before the experiment. ((**Upper**): Cont) The cells were cultured in high-glucose-containing DMEM under normoxic conditions (95% air and 5% CO_2_ at 37 °C) for 15 min. In order to follow the condition of OGD/R stimulation, medium change was performed at 10 min. PBS, a vehicle, was treated throughout the experiment. ((**Middle**): OGD/R) The cells were cultured in glucose-free DMEM under hypoxic conditions (1% O_2_, 94% N_2_ and 5% CO_2_ at 37 °C) for 10 min (OGD). Then, the cells were cultured with high-glucose-containing DMEM in normoxic conditions for 5 min (R). PBS was treated throughout the experiment. (**Lower**) The cells were stimulated with OGD/R. Canstatin (+canstatin: 250 ng/mL), gp91 ds-tat (+gp91 ds-tat: 2.5 µM) or tempol (+tempol: 1 mM) was treated throughout the experiment.

**Figure 4:**
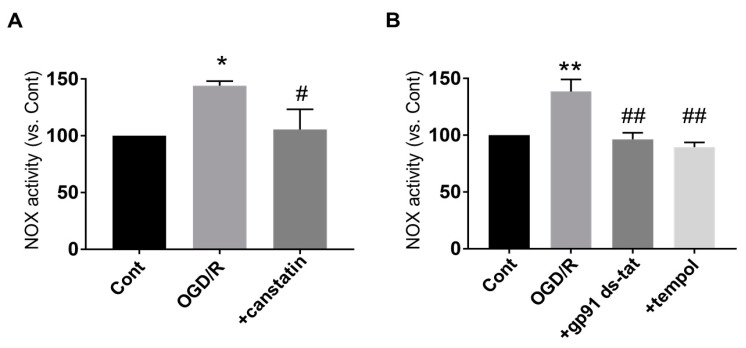
Canstatin inhibited the OGD/R-induced activation of nicotinamide adenine dinucleotide phosphate (NADPH) oxidase (NOX) in NRCMs. NRCMs were stimulated with OGD/R (see the protocols described in Figure 3). Control cells were cultured in the reperfusion medium under normoxic conditions (Cont; (**A**,**B**)). PBS (Cont, OGD/R; (**A**,**B**)), canstatin (+canstatin: 250 ng/mL; (**A**)), gp91 ds-tat (+gp91 ds-tat: 2.5 µM; (**B**)) or tempol (+tempol: 1 mM; (**B**)) was treated throughout the OGD/R stimulation. Total cell lysates of the NRCMs were harvested and NOX activity was determined by lucigenin assay. The normalized NOX activity relative to PBS was shown as mean ± S.E.M. ((**A**): *n* = 6, (**B**): *n* = 4). *, ** *p* < 0.05, 0.01 vs. Cont, #, ## *p* < 0.05, 0.01 vs. OGD/R.

To investigate the detailed mechanisms of the protective effects of canstatin against I/R-induced ventricular arrhythmia, OGD/R stimulation, which mimics I/R injury in vitro, was performed in NRCMs (Figure 3). I/R injury is known to activate NOX, a major source for ROS production, in myocardium [5]. Thus, a lucigenin assay was performed to examine the effect of canstatin on OGD/R-induced NOX activation in NRCMs. OGD/R significantly induced NOX activation (144.2 ± 4.0%, *p* < 0.05 vs. Cont (Figure 3 Upper), *n* = 6), which was significantly inhibited by canstatin (105.6 ± 17.7%, *p* < 0.05 vs. OGD/R, *n* = 6) (Figure 4A). We confirmed that both gp91 ds-tat (2.5 µM), an inhibitor of NOX, and tempol (1 mM), an antioxidant, significantly inhibited the OGD/R-induced NOX activation (gp91 ds-tat: 96.4 ± 5.8%; tempol: 89.6 ± 4.0%, *p* < 0.01 vs. OGD/R, *n* = 4) (Figure 4B).

### 2.3. Canstatin Inhibited OGD/R-Induced ROS Production in NRCMs

**Figure 5:**
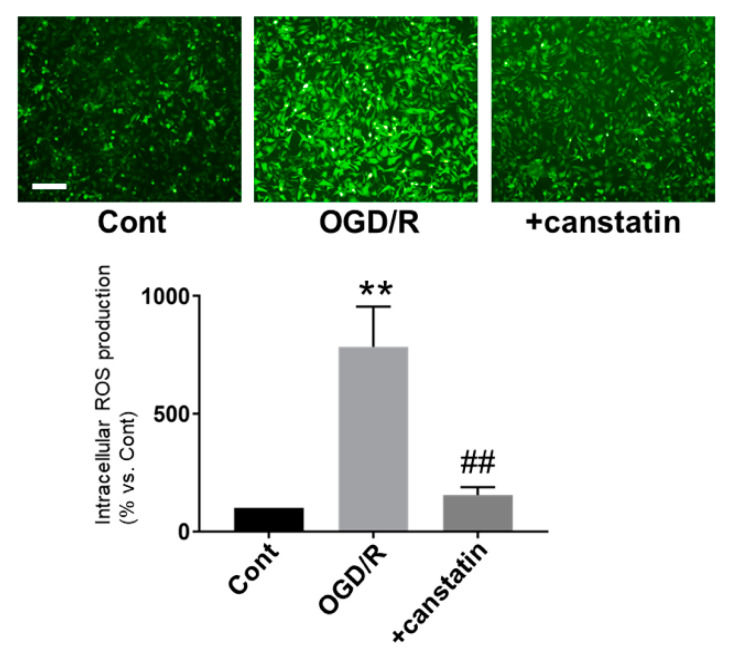
Canstatin inhibited OGD/R-induced reactive oxygen species (ROS) production in NRCMs. NRCMs were stimulated with OGD/R (see the protocols described in Figure 3). Control cells were cultured in the reperfusion medium under normoxic condition (Cont). PBS (Cont, OGD/R) or canstatin (+canstatin: 250 ng/mL) was treated throughout the OGD/R stimulation. The cells were treated with 2′,7′-dichlorodihydrofluorescein diacetate (DCF-DA; 10 µM) for 30 min to detect intracellular ROS production. (**Upper**) Representative images for DCF-DA-stained cells were shown. Scale bar: 100 μm. (**Lower**) The normalized fluorescent intensity of DCF-DA relative to PBS was shown as mean ± S.E.M. (*n* = 6). ** *p* < 0.01 vs. Cont, ## *p* < 0.01 vs. OGD/R.

ROS production is closely associated with I/R-induced arrhythmia [16]. Thus, 2′,7′-dichlorodihydrofluorescein diacetate (DCF-DA) staining was performed to examine the effect of canstatin on OGD/R-induced ROS production in NRCMs. OGD/R significantly induced ROS production (784.5 ± 156.3%, *p* < 0.01 vs. Cont, *n* = 6), which was significantly inhibited by canstatin (155.4 ± 30.1%, *p* < 0.01 vs. OGD/R, *n* = 6) (Figure 5).

### 2.4. Canstatin Inhibited H_2_O_2_-Induced [Ca^2+^]_i_ Rise in NRCMs

**Figure 6:**
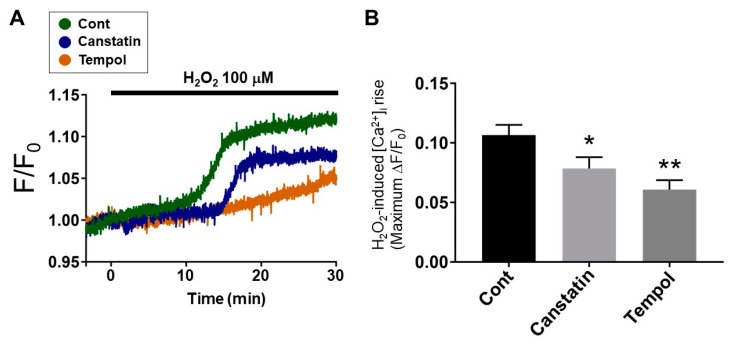
Canstatin inhibited H_2_O_2_-induced intracellular Ca^2+^ ([Ca^2+^]_i_) rise in NRCMs. NRCMs were stimulated with H_2_O_2_ (100 µM) for 30 min following 10 min pre-treatment with PBS, canstatin (250 ng/mL) or tempol (1 mM). [Ca^2+^]_i_ rise was measured by using Fura-2 acetoxymethyl ester, a fluorescent Ca^2+^ indicator. The F340/F380 ratio (F) was calculated and normalized by the basal fluorescence (F_0_) at 30 s before H_2_O_2_ stimulation (F/F_0_). (**A**) Representative time course of F/F_0_ for 30 min in NRCMs stimulated with H_2_O_2_ in the presence of PBS (Cont: Green), canstatin (Blue) or tempol (Brown) was shown. The fluorescence was recorded every 0.1 s. (**B**) The maximum F/F_0_ change (ΔF/F_0_) caused by H_2_O_2_-induced [Ca^2+^]_i_ rise was shown as mean ± S.E.M. (Cont, Canstatin: *n* = 9; tempol: *n* = 7). *, ** *p* < 0.05, 0.01 vs. Cont.

The ROS-induced [Ca^2+^]_i_ rise in cardiomyocytes leads to ventricular arrhythmias [16]. Thus, we investigated the effect of canstatin on [Ca^2+^]_i_ increase induced by H_2_O_2_ (100 µM) in NRCMs. H_2_O_2_ induced a [Ca^2+^]_i_ rise (0.107 ± 0.009), which was significantly inhibited by canstatin (0.079 ± 0.010, *p* < 0.05 vs. Cont, *n* = 9) (Figure 6A,B). Tempol also significantly inhibited the H_2_O_2_-induced [Ca^2+^]_i_ rise (0.061 ± 0.008, *p* < 0.01 vs. Cont, *n* = 7) (Figure 6A,B).

## 3. Discussion

In the present study, we for the first time demonstrated that canstatin suppressed I/R-induced ventricular arrhythmia in rats. Furthermore, canstatin inhibited OGD/R-induced NOX activation and ROS production, and suppressed the H_2_O_2_-induced [Ca^2+^]_i_ rise in NRCMs.

ST elevation is a characteristic waveform observed during ischemia in a rat model of I/R-induced ventricular arrhythmia [17]. In this study, ST elevation by left anterior descending artery (LAD) ligation was confirmed in all rats (Figure 2A). In PBS-administered rats, VT and VF were observed during reperfusion similarly to the previous studies [18,19]. Thus, we successfully made an in vivo model of I/R-induced ventricular arrhythmia. In the present study, canstatin suppressed the occurrence of VF and inhibited the duration of ventricular arrhythmia after reperfusion (Figure 2). NOX is a major source for ROS production in the I/R-injured heart [5]. The ROS mainly contribute to I/R injury [4,5] through the mechanisms including Ca^2+^ overload, which leads to ventricular arrhythmia [20]. In this study, canstatin significantly inhibited the OGD/R-induced NOX activation and ROS production in NRCMs (Figure 4 and Figure 5). Thus, it is suggested that canstatin exerts an anti-arrhythmic effect against I/R stimulation perhaps in part through the inhibition of NOX-induced ROS production and [Ca^2+^]_i_ rise.

The anti-angiogenic and anti-tumor effects of canstatin were mediated through its binding to α_v_β_3_ and α_v_β_5_ integrins [21]. It has been reported that integrins were associated with NOX activation and ROS production [22,23], and that type IV collagen mediated the activation of NOX1 through their binding to α_2_β_1_ integrin in the human adenocarcinoma cell line [22]. The ectodomain of syndecan-4, a heparan sulfate proteoglycan, induced ROS production through the binding to α_v_β_3_ integrin in mouse podocytes [23]. On the other hand, the expression of integrins on the cell membrane was increased by an ischemic stimulation [24,25]. We previously demonstrated that hypoxia induced the recruitment of the α_v_ integrin to the focal adhesion of the cell membrane [26], and that canstatin activated the focal adhesion of kinase/Akt signaling under the hypoxic condition in H9c2 cardiomyoblasts [26]. Thus, it is presumed that the inhibitory effects of canstatin against NOX activation and ROS production might be caused by their binding to integrins.

ROS induce the [Ca^2+^]_i_ rise through the activation of Ca^2+^ handling proteins, such as LTCC, NCX and RyR [6]. In the present study, we demonstrated that canstatin inhibited the H_2_O_2_-induced [Ca^2+^]_i_ rise in NRCMs (Figure 6). We previously reported that the LTCC current was increased in ventricular myocytes from the *COL4A2* siRNA-injected rats, which was reversed by canstatin [14]. In addition, canstatin inhibited the isoproterenol-induced [Ca^2+^]_i_ rise in NRCMs [15]. Thus, it is suggested that canstatin inhibits the ROS-induced [Ca^2+^]_i_ rise by regulating intracellular Ca^2+^ handling. Wu et al. demonstrated that soluble ligands of α_v_β_3_ integrin inhibited the LTCC current in rat arteriolar smooth muscle cells [27]. Furthermore, β_1_ integrin colocalized with RyR in T-tubules protected I/R injury in mouse cardiomyocytes by regulating intracellular Ca^2+^ handling [28]. Thus, it is presumed that canstatin inhibits the ROS-induced activation of Ca^2+^ handling proteins through their binding to integrins.

Verapamil, which was used as a positive control in this study, completely blocked the I/R-induced ventricular arrhythmia in rats (Figure 2A). Although Ca^2+^ channel blockers, including verapamil, are clinically used for the treatment of supraventricular arrhythmias [29], the adverse events, including the worsening of heart failure, have been indicated [9]. It is predicted that canstatin hardly exerts side effects since it is an endogenous peptide abundantly expressed in normal hearts [12]. We previously reported that the long-term administration of canstatin (20 µg/kg/day, 28 days, i.p.) had no effect on cardiac function in normal rats [15,30]. In addition, we recently demonstrated that the administration of canstatin (20 µg/kg/day, 28 days, i.p.) improved survival rate and cardiac dysfunction in MI model rats [30]. Thus, it is suggested that canstatin might exert an anti-arrhythmic effect without exaggerating cardiac dysfunction after MI.

There are limitations to the present study. First, canstatin was administered to rats before I/R injury. However, in AMI patients receiving percutaneous coronary intervention, drug administration is performed at the time of reperfusion. Thus, we should investigate the anti-arrhythmic effects of canstatin at the time of reperfusion to mimic the clinical scenario in a future study. Second, the duration of ischemia protocol in both in vivo and in vitro experimental studies was short, which was sub-lethal. Thus, our results are not able to directly extrapolate into a typical human AMI, which leads to severe cardiomyocyte death and infarction. Thus, we should investigate whether canstatin exerts anti-arrhythmic effects by using an alternative experimental model that is more relevant to the clinical scenario in a future study.

## 4. Materials and Methods

### 4.1. Regents

The reagent sources were as follows: recombinant mouse canstatin (produced by *Escherichia coli* as described previously [15]), verapamil and tempol (Sigma-Aldrich, St. Louis, MO, USA), gp91 ds-tat (Eurogentec, Seraing, Belgium) and H_2_O_2_ (Kanto Chemical, Tokyo, Japan). PBS (NaCl 137 mM, KCl 2.7 mM, Na_2_HPO_4_ 10 mM, KH_2_PO_4_ 1.8 mM, pH 7.4) was used as a vehicle for recombinant canstatin.

### 4.2. Animals

All animal experiments were approved by the President of Kitasato University through the judgement of Institutional Animal Care and Use Committee of Kitasato University (Approval No. 18-019 (18 June 2018), 19-126 (29 August 2019)). Male Wistar rats (CLEA Japan, Tokyo, Japan) were cared in accordance with the guideline for animal care and treatment of the Kitasato University. For the production of an in vivo model of I/R-induced ventricular arrhythmia and the isolation of NRCMs, 10-week-old and 1–3-day-old rats were used, respectively.

### 4.3. In Vivo Model of I/R-Induced Ventricular Arrhythmia

Ten-week-old rats (*n* = 13) were anesthetized with urethane (1.4 g/kg, i.p.). Then, the rats were artificially ventilated (respiratory rate: 80 times/min; tidal volume: 5 mL; SN-480-7; Shinano, Tokyo, Japan) following an endotracheal intubation, and intravenous cannulation to the saphenous vein was performed for the administration of drugs. After a left thoracotomy was performed, 3 Spring Clip Electrodes (MLA1210; AD Instruments, Colorado Springs, CO, USA) connected to a 3-Lead Shielded Bio Amp Cable (MLA2340; AD Instruments) were fixed on the right axillary (cathode), left pectoral (anode) and right inguinal (earth) regions. ECG recording was performed by using a Bio Amp (FE132; AD Instruments) and PowerLab system (ML825; AD Instruments) in lead II configuration. A 6-0 nylon suture was passed under LAD. The suture was threaded through a polyethylene tube to create a snare. Recombinant canstatin (20 µg/kg, *n* = 5), PBS (*n* = 5) or verapamil (0.63 mg/kg, *n* = 3) was intravenously administered for 5 min. Verapamil was used as a positive control. Then, LAD ligation was performed by tightening the snare (ischemia). Ten minutes after the ligation, it was released (reperfusion). VT and VF were recorded for 10 min after reperfusion (Figure 1). The incidence and duration of ventricular arrhythmia during the recording were calculated.

### 4.4. Isolation of NRCMs

NRCMs were isolated from neonatal Wistar rats as described previously [15]. The hearts harvested from 1–3-day-old Wistar rats were washed in PBS with 20 mM 2,3-butanedione monoxime (BDM) on ice. Then, the ventricles of the hearts were minced into small pieces and washed in wash solution (Hank’s Balanced Salt Solution with 0.08% trypsin and 20 mM BDM) for 2 h at 4 °C with stirring followed by an incubation in collagenase solution (Leibovitz’s L15 medium with 0.15% collagenase and 20 mM BDM) for 30 min at 37 °C. The suspension, tissue fragments of which were removed by filtration, was centrifuged at 100× *g* for 5 min at 4 °C, and the pellet was resuspended in high- glucose Dulbecco’s modified Eagle medium (DMEM; Wako, Osaka, Japan) containing 10% feral bovine serum (FBS; Gibco/Lifetechnologies, Carlsbad, CA, USA), 1% antibiotic-antimycotic mixed solution (Nacalai tesque, Kyoto, Japan) and 100 µM bromodeoxyuridine (BrdU). The cell suspension was pre-plated for 90 min twice to remove the attached non-cardiomyocytes. The non-attached cardiomyocytes were collected, seeded and cultured on culture dishes (for lucigenin assay) or coverslips coated with 1% gelatin (for DCF-DA staining or measurement of [Ca^2+^]_i_) in high-glucose DMEM containing 10% FBS, 1% antibiotic-antimycotic mixed solution and 100 µM BrdU.

### 4.5. OGD/R

To mimic I/R injury, OGD/R was performed in NRCMs as described previously [31] (Figure 3). After NRCMs at subconfluence were starved for 24 h in serum-free high-glucose DMEM, the culture medium was replaced with glucose-free DMEM (Wako) under hypoxic conditions (1% O_2_, 94% N_2_ and 5% CO_2_ at 37 °C) in the multi-gas incubator (BL-42MD; JUJI field Inc., Tokyo, Japan) for 10 min (OGD). Then, the cells were cultured with the high-glucose DMEM under normoxic conditions (95% air and 5% CO_2_ at 37 °C) for 5 min (R). Recombinant canstatin, PBS, gp91 ds-tat or tempol were treated throughout the experiment. Control cells were cultured in high-glucose-containing DMEM under normoxic conditions. After the OGD/R stimulation, the cells were used for lucigenin assay or DCF-DA staining.

### 4.6. Lucigenin Assay

To assess the activity of NOX in NRCMs, a lucigenin assay was performed as described previously [32]. Total cell lysates were harvested by lysis buffer (Nacalai tesque). Phosphate buffer (200 µL: 50 mM NaH_2_PO_4_ and NaHPO_4_, 1 mM EGTA and 150 mM sucrose at pH 7.0) containing lucigenin (10 µM), NADPH (1 mM) and cell lysate (20 µg) was poured into assay wells (96-well plates). Then, the chemiluminescence was continuously measured for 30 min at 37 °C by a TriStar LB941 luminometer (Berthold, Bad, Wildbad, Germany). The chemiluminescence of relative light units per second (RLU/s) was obtained every 10 s, and the results were calculated as area under the curve.

### 4.7. DCF-DA Staining

To evaluate intracellular ROS production in NRCMs, DCF-DA staining was performed as described previously [31]. The cells were incubated with DCF-DA (10 µM; Invitrogen, Carlsbad, CA, USA) for 30 min at 37 °C. Fluorescence images were obtained by a microscope digital camera (DP-74; OLYMPUS, Tokyo, Japan)-equipped fluorescent microscope (BX-51; OLYMPUS). The fluorescent intensity was measured by Image J software (Version 1.52a; National Institutes of Health, Bethesda, MD, USA).

### 4.8. Measurement of [Ca^2+^]_i_ in NRCMs

[Ca^2+^]_i_ in NRCMs was measured by using Fura-2 acetoxymethyl ester (AM) (Nacalai tesque) as described previously [15]. The cells were incubated with normal 4-(2-Hydroxyethyl)-1-piperazineethanesulfonic acid (HEPES)-Tyrode solution (1.8 mM CaCl_2_, 143 mM NaCl, 5.4 mM KCl, 0.33 mM NaH_2_PO_4_, 0.5 mM MgCl_2_ 6H_2_O, 5.5 mM Glucose and 5 mM HEPES) with Fura-2 AM (5 μM) for 30 min at 37 °C, and then incubated with Fura-2 AM-free normal HEPES-Tyrode solution for 30 min at 37 °C. Then, the cells were alternately excited at 340 and 380 nm by using a rotating filter wheel, and the fluorescence (emissions at 500 nm) of Fura-2AM was obtained by a dual-wavelength fluorometer (CAM-230; Japan Spectroscopic Co, Ltd., Tokyo, Japan). NRCMs were stimulated with H_2_O_2_ (100 µM, 30 min) after pretreatment with PBS, canstatin (250 ng/mL) or tempol (1 mM; positive control) for 5 min. The F340/F380 ratio (F) was calculated and normalized by the basal fluorescence (F_0_) at 30 s, obtained before H_2_O_2_ treatment.

### 4.9. Statistical Analysis

Data are presented as mean ± standard error of the mean (S.E.M.). In two group comparisons, statistical analyses were performed by unpaired two-tailed Student’s *t*-test (Figure 2B). In the multi-group comparison, statistical analyses were performed by one-way ANOVA followed by Bonferroni’s post hoc test (Figure 4A,B, Figure 5 and Figure 6B). A value of *p* < 0.05 was considered statistically significant.

## 5. Conclusions

Our data suggest that canstatin is preventive against I/R-induced ventricular arrhythmia, perhaps in part through the suppression of ROS production and the subsequent [Ca^2+^]_i_ rise in cardiomyocytes. It is expected that canstatin contributes to the development of a novel therapeutic strategy to suppress I/R-induced ventricular arrhythmia in AMI patients.

## Data Availability

The datasets presented in this study are available from the corresponding author upon reasonable request.
